# Confessing in the GDR. The Psychology of Interrogation under Socialist Rule, 1961–1989

**DOI:** 10.1007/s00048-025-00433-5

**Published:** 2025-10-28

**Authors:** Martin Wieser

**Affiliations:** 1 Sigmund Freud PrivatUniversität Berlin, Columbiadamm 10, Turm 9, 12101 Berlin, Germany; 2 Sigmund Freud Privatuniversität Wien, Wien, Österreich

**Keywords:** Interrogation, Criminology, GDR, Socialism, Forensic psychology, History of psychology, Vernehmung, Verhör, Kriminalistik, DDR, Sozialismus, Forensische Psychologie, Geschichte der Psychologie

## Abstract

This article provides a historical analysis of the institutional, legal, and psychological foundations of the study of interrogation in the post-Stalinist GDR. Drawing on archival and published documents, the development of the Criminological Section and Forensic Psychology at Humboldt University from the 1960s to the early 1990s is reconstructed. After giving an overview of the institutional context and the main actors in this field, the analysis highlights the relevance of psychological knowledge from the perspective of GDR criminologists. These aspects include the preparation phase, the initial contact with the defendant, the continuous dissection of statements during the interrogation, and the overarching goal of gaining a complete picture of the suspect’s personality. It is argued that the defining feature of interrogation practice in the GDR was not so much the technical details as the overarching aim of “educating” the suspect, who was to be persuaded by the interrogator not only to confess to the crime, but also to show remorse and find his way back into socialism.

## Abstract (de)

Der Beitrag liefert eine historische Analyse der institutionellen, rechtlichen und psychologischen Grundlagen der Vernehmungslehre in der poststalinistischen DDR. Auf der Grundlage von archivierten und publizierten Dokumenten wird die Entwicklung der Sektion für Kriminologie und der Forensischen Psychologie an der Humboldt-Universität von den 1960er bis zu den frühen 1990er Jahren rekonstruiert. Nach einem Überblick über den institutionellen Kontext und die zentralen Akteure wird die Relevanz psychologischen Wissens in verschiedenen Phasen des Vernehmungsprozesses aus der Sicht der DDR-Kriminologen herausgearbeitet. Zu diesen Aspekten gehören die Vorbereitungsphase, der Erstkontakt mit dem Beschuldigten, die kontinuierliche Bewertung von Aussagen während der Vernehmung und das übergeordnete Ziel, ein vollständiges Bild der Persönlichkeit des Verdächtigen zu gewinnen. Wie gezeigt wird, liegt das bestimmende Merkmal der Vernehmungspraxis in der DDR weniger in den technischen Details als in dem übergeordneten Ziel der „Erziehung“ des Verdächtigen, welcher durch den Vernehmer nicht nur zu einem Geständnis der Tat, sondern auch zur Reue und Rückkehr in den Sozialismus angeleitet werden sollte.

## Confessing in the GDR: The Study of Interrogation under Socialist Rule, 1961–1989

“Rubashov was silent. Quite a long time passed. Ivanov’s head bent even closer over the writing desk. ‘I don’t understand you,’ he said. ‘Half an hour ago you made me a speech full of the most impassioned attacks against our policy, any fraction of which would have been enough to finish you off. And now you deny such a simple logical deduction as that you belonged to an oppositional group, for which, in any case, we hold all the proofs.’

‘Really?’ said Rubashov. ‘If you have all the proofs, why do you need my confession?’” (Koestler 1941: 89)

Did socialists interrogate differently? Following Antje Schumann’s definition of interrogation as an act of “asymmetrical communication” that is located at the “intersection” of the state and the citizen (2016: 1), the answer seems obvious: if there ever existed a substantial difference between the structure of the socialist states and their liberal counterparts, this divergence somehow must have been reflected in the application of interrogation practices. This differentiation between democratic and socialist modes of governance was called into question by Michel Foucault, who argued in his lectures on biopolitics at the Collège de France that there existed no “autonomous governmentality of socialism.” From Foucault’s perspective, socialist states incorporated “liberal governmentalities” to varying degrees, along with “hyper-administrative” or “police state” practices of governance (Foucault 2008: 29). However, Foucault never specifically addressed socialist practices of criminal interrogation in his analyses, and the existing historical literature concerned with the development of interrogation practices that has been published in the meantime is almost exclusively concerned with their development in Europe and North America (Niehaus 2003; Reichertz & Schneider 2007; Schumann 2016). As of today, many aspects of the roots and development of interrogation practices as techniques of socialist governance are still as much in the dark as they were when Foucault held his lectures on biopolitics in the late 1970s.

Taking up a historical angle, our initial question leads us back to the dawn of the Soviet era. In the infamous “Moscow trials” that took place under Stalin’s rule between 1936 and 1938, a number of high-profile members of the Communist Party were accused of counter-revolutionary activities. After several months in prison, the defendants publicly confessed to betraying the communist revolution by conspiring with hostile forces. For the public, these show trials revealed an unexpected willingness by the old Bolsheviks to confess to even the most absurd conspiracies. Their conviction sparked speculations about the events preceding these confessions: Was it mere physical violence and torture that broke their will, or was it something else? And why would they not use their last chance in public to defend themselves as they faced their inevitable death?

The Hungarian-Austrian Arthur Koestler was one of the first authors to publicly address these questions, albeit in a semi-fictional way. His novel *Darkness at Noon*, first published in 1941, recounts the final days of the fictional protagonist Rubashov, a revolutionary of the first days. Rubashov climbed up the ranks of the Party after its victory, until he finally became a victim of the apparatus he had helped to construct. From the prison cell to the interrogation room, the protagonist is repeatedly confronted with his former comrades, who are now in charge of his prosecution. In Koestler’s narrative, the interrogation of Rubashov represents much more than a mere criminal procedure: it is portrayed as a stage where the old guards of the revolution and the young apparatchiks face off, exchanging philosophical, historical, and moral arguments about whether the life of one individual can be weighed against a superordinate idea and the supposedly inevitable progress of history. Rubashov denies any allegations of conspiring against the dictator, but he openly shares his doubts about the moral justification of a worldview that sees any means as justified to achieve a “higher” end. Finally, Rubashov’s opponents convince him to give up the pointless fight and render his last service to the Party. After several weeks of isolation and interrogation, Rubashov confesses to the alleged conspiracy and is thereafter sentenced to death by a shot in the head.

The scenery of *Darkness at Noon* is partly based on the author’s own biography. As a member of the German Communist Party since 1932, Koestler visited the Soviet Union in the early 1930s. After the Spanish Civil War broke out, Koestler travelled there as a war reporter, only to be captured, detained, and sentenced to death by the Spanish Nationalists. Diplomatic intervention by the British saved Koestler’s life, and immediately after his release he began working on the manuscript of his novel. Koestler’s book was not just inspired by his personal experience of imprisonment, but also bore witness to his departure from the Communist Party in the face of millions of victims during the “Red Terror” under Stalin. At the dawn of the Cold War, Koestler’s book had a significant influence on the European left, comparable only to Solzhenitsyn’s *Gulag Archipelago*, which was published thirty years later. While Solzhenitsyn’s story illustrates the hopelessness and defenselessness of the inmate in the labor camp, Koestler’s novel focuses on the power dynamics that unfold between the prison cell and the interrogation room. According to Koestler, his book inspired the so-called “Rubashov-theory of confessions” (Koestler 1979: 237), arguing that the outcome of the Moscow trials was not merely a consequence of physical violence or torture. Instead, the old guards of the revolution confessed because the interrogators made them believe that they had only one option left: to present their fate as a warning to all present and future enemies of the socialist state.

Three years after Stalin’s unexpected death in 1953, the infamous secret speech of his successor Nikita Khrushchev at the 20th Congress of the Communist Party denounced the “cult of personality” installed by Stalin and the excesses of violence during the “purges” of the 1930s. The “Khrushchev thaw” brought structural reforms to the Soviet system on many levels, including a decline in public uses of violence and show trials, a partial easing of censorship in public life and the press, the deconstruction of the Gulag System, and a release of thousands of political prisoners from labor camps and prisons. In the following decades, state socialism went through several phases of partial liberalization and recurrent political repression, which took different courses in different parts of the Soviet sphere until its final collapse in the early 1990s. State violence, politicized jurisdiction, censorship, and repression never disappeared in post-Stalinism, but their form and intensity were subject to fundamental change. The Moscow trials were not reenacted after Stalin’s death, but this does not mean that the practice of interrogation as a means to exert political power totally disappeared, even though the use of physical violence during detainment declined.

As I argue in the following essay, the interrogation of suspects retained its central position at the intersection of politics and science, police work and the juridical system in post-Stalinism and late socialism. Using the example of the GDR during and after the 1960s, I will discuss how practices of interrogation evolved into a matter of concern for a growing number of experts and professions. Politicians, legal scholars, police and intelligence officers, criminologists as well as psychologists contributed to a growing body of knowledge that addressed the uses and function of interrogation, aiming to refine its techniques, broaden its scope, and increase its efficiency. Based on historical literature, published material, and archival sources from the former GDR, I aim to highlight the genesis and development of interrogation knowledge from the 1960s until the late 1980s. With a special focus on the role of forensic psychology and criminology, the aim is to provide a coherent picture of the “socialist interrogator”, as constructed by criminological experts.

After providing a brief overview of the existing historical literature on interrogation and detention in the GDR, I will focus on one singular institution that fulfilled a central function in the production of knowledge on interrogation in the GDR: the Criminological Section of Humboldt University Berlin. Hundreds of members of the police, the military, customs, and the interior ministry were sent to this institution from the 1960s onwards, which also had strong links with the secret service of the GDR (Kowalczuk 2012). While many documents created and used at the Criminological Section were subject to confidentiality, some of them were also accessible to a broader audience and other experts within the executive and judiciary systems, such as police officers, judges, and public prosecutors. To professionalize the planning and conduct of interrogations in the post-Stalinist era, psychologists and legal experts were tasked with collecting and evaluating interrogation knowledge from both the East and West. Based on their analysis, they aimed to identify and improve the interrogators’ practices and their training. In the following, I will first provide an overview of the institutional development of the Criminological Section at Humboldt University. Building upon that, the legal and epistemological foundations of interrogation knowledge in the GDR will be summarized, followed by the central steps of planning and conducting interrogations, as described in criminological literature. Finally, I will discuss the historical relevance of socialist interrogation knowledge at the intersection of political, legal, and scientific concerns, as well as the tensions and ambivalences that arose between these professional fields in the GDR.

## Interrogation and Detention in the GDR: Sources and Perspectives

A number of historical studies have addressed the development and contexts of the detention system in the GDR (Engelmann & Vollnhals 1999; Selitrenny 2003; Passens 2012; Martin 2014; Spohr 2015). Most of these works draw heavily on the archives of the Ministry of State Security and its detention centers, which were made accessible to the public after the reunification of Germany in the early 1990s. All of these studies shed light on the role of pre-trial detention within the overall justice system as well as the internal regulations and social structure of the detention system in East Germany. From these analyses, we learn about the reasoning behind the architecture of the prisons and their development during and after the Stalinist era, the regulations that structured daily routines inside the prison, the rules of conduct for prisoners, how secret informants were used to gather information from inside the prison, as well as the social background and training of the interrogators and the prison guards. All of these works agree that the GDR’s prison system went through a number of transformations during the 1960s. These changes included the decrease of physical abuse during detainment, the systematic use of solitary confinement and long overnight interrogations, especially during the first days after the arrest, frequent rotation of interrogators, the use of protocols as a means to prepare the indictment (Sälter 2011) and, most importantly, the overall goal of this whole ensemble of material and social elements, namely to increase pressure on the defendant to confess (Gieseke 2014: 140).

Through this extensive body of historical literature, the significant role of interrogation practices in the detention system and the wider political context of East Germany under socialist rule has been brought to light. However, the interpersonal, communicative, emotional, and social dynamics that materialized between the interrogator and the defendant as well as the sources of knowledge that were used in order to control these factors have been discussed so far only on the periphery. Archival documents, such as interrogation plans or protocols “which sometimes appear to be verbatim transcripts, but which in fact have the character of reports by the interrogator” (Engelmann 1995: 254), are usually of limited value for this purpose, since they primarily represent the perspective of the interrogators and their anticipated use of these documents in court. Stasi files, as the historian Jens Gieseke emphasized, represent a “record of police bureaucracy, which should not be confused with the events represented by them” (Gieseke 2019: 91). Other sources can be used to fill this gap, such as reports of former political prisoners who testified about their time in custody after their escape or release to the West (Hetmann 1961; Bundesministerium für gesamtdeutsche Fragen 1962; Lolland & Rödiger 1977; Fuchs 1978). Autobiographical accounts offer a vivid and compelling view from inside the prison, the shock of arrest, the grueling life behind prison bars, and the endless confrontations with interrogators. However, it is usually very difficult to verify their accuracy, especially if no other documents are available for comparison. The extreme stress experienced after arrest and the monotonous nature of prison life make it very challenging to maintain an accurate memory of the sequence of events. In addition, reports that were published before 1989 often had to be anonymized and redacted in order to protect the authors and their friends and families. Reports from political prisoners that were published after the reunification of Germany (Fritzsch 1993; Jablonski 1997; Knabe 2007) were no longer subject to these restrictions, but they suffered from a greater time lag, which presumably made them more susceptible to distortions of memory. Since many details about the practices of the State Security had become public in the meantime, common knowledge also could have influenced these individual reports in various ways.

Thanks to the manifold written and oral sources available today, the practice of interrogation in the GDR can be analyzed from various perspectives. In the following analysis, I will approach this topic from a discursive and historical angle, aiming to reconstruct socialist interrogation knowledge by presenting documents that were created to analyze, systematize, and improve practices of interrogation in the GDR by experts from psychology, criminology, law, and the secret service. Admittedly, it cannot be assumed that these documents provide clear evidence about individual cases. However, they can be read as normative sources that inform us how psychologists, criminologists, and officers portrayed the “ideal” course of interrogations, which problems they identified, and how they aimed to increase the effectiveness of interrogation practices.

## The Establishment of Criminology at Humboldt University

As part of the training of law students, criminology entered the German academic landscape in the late nineteenth century. Legal reformers such as Hans Gross and Franz von Liszt promoted the introduction of courses from anthropology, biology, sociology, psychiatry, and psychology into the training of legal and criminal experts (Wetzell 2000). In his seminal introduction to criminal psychology, Gross argued that “we have to admit that we never treated law as a science, we have never regarded it as an empirical science. […] As scientific legitimization, we need above all to include research disciplines that have a direct connection with our work” (Gross 1905: 7). After World War I, criminal psychology became a regular topic in the training of legal experts and the police (see also Hofman and Schlicht, this issue), competing with biologically oriented anthropologists and psychiatrists, such as Cesare Lombroso. The practical scope of criminal psychology was mostly limited to the examination of testimonies, the assessment of the credibility of witnesses, and characterological examinations of defendants or witnesses (Mülberger 2009; Wolffram 2018). Expert opinions on psychopathological issues were routinely provided by forensic psychiatrists only. Despite these continued efforts to increase the proportion of empirical and practical knowledge in the study of law, forensic psychology did not become an independent academic field in Germany before World War II.

In the GDR, the institutionalization of forensic psychology was preceded by the establishment of the Institute of Criminology as part of the Faculty of Law at Humboldt University in August 1951. Despite this first step towards official recognition of criminology in the young socialist state, the members of the institute remained on the margins of the Faculty of Law during its first decade of existence. Counting only one professor (a trained pharmacist) and half a dozen assistants with little practical criminological expertise, members of the Institute of Criminology could only offer a few introductory courses covering the basics of criminology for students of law. Their practical activities were mostly concerned with the provision of technical assistance in criminal investigations, such as the examination of traces at crime scenes or the analysis of handwriting or forged documents (Schurich & Wirth 2015: 39). Up to this point, criminology represented more of an auxiliary assemblage of diverse practices and apparatuses than a distinct scientific discipline. Among the early members of the institute, there were no designated experts from psychology or psychiatry.

It took several more years until criminologists managed to step out of the fringes of the Faculty of Law. In 1958, criminological departments were established in Jena, Leipzig, and Halle, where criminological courses were offered to law students. Two years later, Ehrenfried Stelzer, a lawyer by training, and a member of the criminal police and the secret service, was appointed director of the Institute of Criminology at Humboldt University Berlin. In October 1961, he was appointed head of the “subject area” [*Fachrichtung*] of criminology. Under Stelzer’s leadership, the small but eager group of GDR criminologists first turned towards the Soviet Union, where criminology was widely recognized as a scientific discipline in its own right, travelling to Moscow in the late 1950s and visiting several criminological institutes across the Soviet Union. Back in Berlin, they translated several criminological works from Russian to German, such as Koldin’s *Theory and practice of criminalist identification* (1959) or Mitritchev and Tersijew’s introduction to criminology (Stelzer & Stelzer 1961). Specialized literature was also made available to the German-speaking audience, such as *The Interrogation* (Stelzer & Stelzer 1960), *Investigative Tactics in Soviet Criminology *(Wassiljew 1962), or *Forensic Psychology for Investigators* (Ratinow 1970)—one of the most widely cited books in this field over the next three decades.

In January 1962, the first group of students was admitted to the criminological study program at Humboldt University.^1^ Initially, this program was organized by two departments: “Criminology and Forensic Psychology” on the one hand, and “Scientific Court Expertise” on the other. Their members organized a four-year distance study program for about 150 students per year. However, internal documents reveal that Stelzer’s plans went much further than that: the existing two departments were to be expanded to four, one of which was to be dedicated exclusively to the field of “Forensic Psychology and Interrogation Studies.”^2^ Even though these ambitious plans could not be realized as quickly as initially planned, GDR criminology went through a remarkable growth in terms of staff numbers and students in the following years, counting eight professors, seven lecturers, 27 scientific assistants, and more than 500 students by the end of the 1970s.^3^ By 1981, more than 1,400 diploma criminologists had completed their studies at Humboldt University.^4^ One significant reason for this rapid institutional expansion (which was unparalleled in West Germany) was that the vast majority of staff members and students were funded and delegated by state authorities. Students were preselected by the Ministry of the Interior, the customs administration, or the Ministry of State Security. In the first two rounds of matriculation of the on-site study program (which was introduced in 1967), all 53 direct students were delegated by the secret service. In the following years, students were also sent by the customs administration and the Ministry of the Interior, and even a small group of students from Vietnam (who came by a contractual agreement between Vietnam and the GDR) joined the study program. The secret service consistently remained at the top of the list, delegating more than 60 percent of the total number of students until 1980.^5^ Although the existence of the Institute of Criminology was no secret, the true affiliation of its staff members and students was. All of them were somehow connected to the criminal police and most of them also to the secret service (Kowalczuk 2012). Archival records also show that there was also a close collaboration between the Criminological Section and the Juridical Academy of the Ministry of State Security in Potsdam, the major training center for higher members of the Stasi (Wieser 2022). Students and teaching staff were frequently exchanged between both institutions, and many diploma theses written at Humboldt University were supervised by teaching staff from Potsdam (Freese & Maercker 2021). Department IX of the Ministry of State Security, which was responsible for investigation and interrogation, employed a particularly large number of graduates from criminology (Engelmann & Joestel 2016). At the infamous Berlin-Hohenschönhausen detention center, almost 50 percent of the members of the department were graduates from Humboldt University by the end of the 1980s (Martin 2014: 146).

In the course of the state-wide third reform of the higher education system from 1968 (Lambrecht 2007), the Institute of Criminology was detached from the Faculty of Law and transformed into an independent *Sektion* with its distinct study program. The newly introduced four-year direct study program of criminology covered a wide range of topics, including Marxism-Leninism, penal law, mathematics, psychology, psychiatry, medicine, cybernetics, and microelectronics, but also practice-oriented subjects such as criminal tactics, interrogation, and leadership skills. In addition to these courses, students had to write a diploma thesis and complete several internships before they were awarded the title of “diploma criminologist.”^6^ In addition to on-site and distant study programs, a two-year “top-up” study program of criminology was offered for graduates of the Academy of the Ministry of the Interior. Besides teaching, staff members carried out a variety of tasks for the state and the police, such as providing expert opinions, assessing the credibility of witnesses and their testimonies, and developing new methods for the investigation of crime scenes. The majority of the instruction material and diploma theses that were written at the Criminological Section were not available to the public. Nevertheless, members of the Section also published on many different aspects of criminology, sometimes even together with academic psychologists (Hiebsch & Werner 1968; Werner 1978; Werner & Römer 1973), and gave public talks at meetings of the Association of Psychology of the GDR. Two criminological journals, the *Forum der Kriminalistik* (1965–1990) and the *Kriminalistik und forensische Wissenschaften* (1970–1996) were edited by members of the Section, who also published several encyclopedias, handbooks, and monographies on specific criminological topics (an extensive bibliography can be found in Schurich & Wirth 2015: 303–456). Until the dissolution of the Section in the mid-1990s, 37 habilitation theses, 112 dissertation theses, and several hundred diploma theses were finished and about 3,000 students had completed their training at the Criminological Section (Schurich & Wirth 2015: 145).

## The Short Life of Forensic Psychology in the GDR

While GDR criminology continuously expanded on an institutional level, its representatives also aimed to extend the scope of their practical expertise and strengthen the theoretical foundation of their work. Archival records and publications indicate that psychology was assigned a central place in the development of criminology from the 1950s onwards, even though it was often not clear where and how psychological knowledge should be implemented in the departments and their curricula. Besides Ehrenfried Stelzer, who served as the Section’s director since its establishment in 1968, the second key figure was his deputy Axel Römer. As a doctor of law with a specialization in the psychology of testimony and interrogation, Römer joined the Faculty of Law as early as in 1962. From then on, Römer gave courses on criminal psychology and the psychology of interrogation. His engagement in this field effectively made him one of the leading figures in the establishment of forensic psychology in the GDR, even though he had never earned any degree as a psychologist.^7^ In 1969, the first trained psychologist entered the Section: Rolf-Reiner Werner, who previously served as director of a special home for children and teenagers with behavioral problems, was appointed honorary professor in 1969. His appointment was supported by the Ministry of the Interior, as Werner was consulted by the Ministry during the preliminary planning stages for the establishment of the Department of Forensic Psychology. Two years later, after the official introduction of the subject area of Forensic Psychology, Werner became full professor in September 1971. Werner was assigned to teach the whole field of forensic psychology, including the assessments of testimonies, personality psychology, and diagnostics, while his slightly older colleague Axel Römer was assigned to cover “special criminal tactics” and the study of interrogation.

Within this institutional context, representatives of forensic psychology did not just offer individual courses on psychological topics for students of criminology, but also attempted to cultivate their own academic garden. Under the auspices of Reiner Werner, a study program for forensic psychology was established in 1972. The curriculum was conceived as a specialized training for students of psychology who had already completed two years of study before specializing in forensics. After their transfer to the Criminological Section, these students had to complete two more years of courses covering psychological diagnostics, forensic psychiatry, anatomy and physiology, psychotherapy, criminal law, and interrogation tactics, and complete internships on credibility diagnostics.^8^ The program was completed with a thesis and the award of a diploma. Fifteen students were to be admitted to the study program per year. From this group, three students would be allowed to enter a subsequent three-year “research study program” to earn their doctorate.^9^

Unlike criminology, the life span of forensic psychology at Humboldt University was very short. The study program was discontinued only two years after its establishment. One major reason for the quick dissolution of the study program was repeated disciplinary difficulties with its head Werner, who was involved in a number of conflicts with staff members, colleagues, and students. Werner was accused of teaching too unsystematically, of lacking leadership skills, of showing disinterest and aggressive behavior towards his colleagues and supervisors, as well as of drawing on “outdated” and “biologistic” Freudian concepts.^10^ Some of his co-workers resigned because they found him incapable of accepting any professional critique and unwilling to abide by joint agreements.^11^ Beyond personal conflicts, broader issues also played an important role in the dismantling of the study program. Werner promoted a very broad outline of forensic psychology, portraying it as “one of the oldest partners of law” (Werner 1978: 11) that supposedly covers all psychological aspects of the juridical sphere, from the prevention and explanation of criminal behavior to the rehabilitation of offenders (Werner & Römer 1973, Werner 1978). Werner’s professional activities were mostly limited to questions of developmental psychology and personality diagnostics, the psychopathology of criminal behavior, and the resocialization of offenders. Protocols of meetings of the Criminological Section show that Werner’s colleagues repeatedly reminded him that “teaching must be focused on what is necessary in practice.”^12^ Even though his colleagues could “not fully endorse Werner’s view of basic research,”^13^ he insisted that forensic psychology should be acknowledged as a distinct subdiscipline of psychology with its own theoretical and methodological fundament. Werner made no secret of the fact that his ultimate goal was to establish an independent academic and professional field, rather than to provide auxiliary instruments for criminologists. In his view, it was the duty of his juridically trained colleague Axel Römer to offer practical knowledge to the criminological audience, especially with regard to interrogation.^14^ In addition to these converging ideas about the status of forensic psychology, there was no clear consensus on where forensic psychologists should be employed after graduation. Werner was removed from his position as leader of the study program by the end of 1973, but he was able to avert his full dismissal from the Criminological Section thanks to the support of influential spokesmen in the Ministry. His group was reorganized three years later as a “working group” for “criminological psychology” with four members who worked under the leadership of Axel Römer.^15^ From then on, forensic psychology remained a part of the curriculum of criminology, but it was no longer taught to psychologists. In 1995, the whole Criminological Section was closed down for good.

Among the wide range of technical, legal, and psychological topics that were taught and researched at the Criminological Section, the field of interrogation studies had a special significance, despite the fact that Werner contributed very little to it. It was mostly practitioners from law and police work, such as Axel Römer, who argued that interrogation studies should hold a “central position” (Römer 1967: 43) in criminology. Based on an analysis of about 40 selected dissertations and 60 publications from the Criminological Section, all dealing with various theoretical and practical aspects of interrogation, I will now present a thematic overview of the core themes of this body of literature from 1964 to 1990.

## Normative Principles of Socialist Interrogation: Legal and Epistemological Foundations

The common starting point for most works on interrogation was the Code of Criminal Procedure of the GDR. In its 1968 version, under the title “Establishing the Truth,” Paragraph 8 states that all authorities involved in criminal investigations are obliged to examine the “causes and conditions” of a crime from “all perspectives” and in an “impartial” manner. The investigator was responsible for determining the nature of the offense and assessing the defendant’s personality and criminal liability. According to Paragraph 24, reports by suspects or defendants were classified as primary evidence for the investigation, along with the testimonies of witnesses and expert opinions (Ministerium der Justiz 1968: 9). Defendants had the right to refuse to testify, and they were encouraged (but not obligated) to help solve crimes. In the Code of Criminal Procedure, the interrogation of defendants only represented one element in an interconnected chain of legal practices (often referred to as “investigative action”), designed to gather and connect all relevant criminal evidence. Investigations could be initiated by observations made by the authorities, a self-report by the offender, or the statement of a witness. If the initial suspicion was confirmed by the police and a warrant was issued by the prosecutor, the suspect was to be arrested. After their personal identification, the accused were taken to the detention center, where the authorities informed them about the charges against them.

According to the Code, the interrogator sets up a protocol as soon as the interrogation begins. At the end of every session, the protocol had to be checked and signed by the defendant, page by page. If deemed necessary, the detainee could be placed in solitary confinement between the interrogations. Officially, the maximum duration of the preliminary investigation was three months, but it could be extended without prior limit at the request of the prosecutor or the investigating authority (Passens 2012: 47). In Berlin-Hohenschönhausen the average length of detention was about four months, but in extreme cases it could last up to several years (Spohr 2015: 207). More specific rules concerning the rights and duties of prisoners during pre-trial detention were laid down in the detention center’s rules for detainees, including the right to get some fresh air daily and to contact their lawyer and family members. However, the rights of prisoners were often formulated so vaguely that the prison administration could grant or deny “privileges,” such as access to cigarettes or books, at any time. Depending on the type of the alleged crime, different branches of the criminal investigation department could be assigned to lead the investigation. In the case of political crimes, Department IX of the Ministry of State Security was usually in charge of leading the investigation.

Several criminological works placed great hope in the use of psychological knowledge for all aspects of the criminal investigation. Soviet criminologists argued that “the further development of tactical interrogation procedures is inconceivable without the extensive use of psychological knowledge,” and it was the duty of “psychologists, legal scholars and investigative bodies to contribute to the development of speciality areas of psychology—the process formation of statements and the psychology of interrogation” (Stelzer 1960: 17). The criminal law should not be misrepresented by the interrogator as an obstacle to his work, but as a necessary tool to guide and structure his practice. Socialist law, science, and practice must not stand in each other’s way, but should converge and form a unity, helping the interrogator to “make clever use of all tactical means available under the law” (ibid.: 15). Following the Soviet model, GDR criminologists promoted a similar view: a lecture manuscript from the Criminological Section dedicated to the “Concept, Task and Meaning of Interrogation” stated that the legal basis of the Criminal Procedure Code facilitates the “optimal organization”^16^ of the investigation. However, this required not just legal, but also scientific expertise. While the law states that defendants are not obliged to testify, the experienced criminologist knows that most defendants will not completely refuse to make a statement, as this would deprive them of the opportunity to defend themselves against the accusations raised against them. Finding the best way to take advantage of this behavior and guide the conduct of the defendant is primarily a practical, not a legal question, and it was one science in particular that the interrogator should know about: any “modern theory of interrogation that is to meet today’s requirements can no longer be practiced without psychology.”^17^ However, the interrogation only represents one element among several others in the investigative procedure. Whether defendants give a confession or not, their testimony should never be misrepresented as the “crown of proofs” that renders any further investigation obsolete (Stelzer 1960: 68). The law makes no distinction between different types of criminal evidence, and every piece of information must be verified by other available sources.

Beyond the legal framework and the necessity of scientific expertise, further principles and guidelines for criminological investigations were frequently mentioned in GDR criminology, principles which were supposedly derived from the philosophy of Marxism-Leninism. “Objectivity” and “veracity” refer to the requirement of completeness, realism, comprehensiveness, and scientific rigor of the investigation process, which was supposed to be sharply distinguished from the Western, bourgeois, idealist, and subjectivist understanding of reality and human perception. In Marxism-Leninism, the ability to “reflect” the material reality by human consciousness is understood as the fundament of perception, thinking, and remembering. According to Engel’s and Lenin’s “reflection theory,” there is a clearly determinable, objective truth that can be analyzed through scientific rigor. In the investigative process, this search for unambiguous truth represents the guiding principle of the criminologist: “Unity, concreteness and natural as well as logical conditionality of all facts determine their objective nature, which must be the fundament of planning” (Stelzer 1984: 26). “Partiality,” on the other hand, is not understood as a contradiction to objectivity, but as its precondition. The criminological expert is not just a representative of the police, but also represents the socialist state and its law, as well as the socialist society as a whole, which he has vowed to protect and defend (Stelzer 1978: 82). Since every crime represents a threat to the socialist state, the police has the legal and moral duty to solve it and prevent its reoccurrence in the future. Since objective reasons for crimes such as robbery or fraud officially did not exist in the socialist state, the criminologist must find their true cause in the remnants of the outdated capitalist society, or in attempts by the class enemy to exert their hostile influence. Unlike the “bourgeois” jurisprudence of capitalism, socialist law does not assume the inevitability of criminal behavior, but emphasizes the possibility of betterment through education.

One special feature of Soviet criminology was the method of “criminological version building.” In essence, this method represented a procedure for systematically formulating empirically testable hypotheses to “ascend and advance from uncertainty to certainty” (Stelzer 1978: 154). The creation of “versions” is defined as a logical intellectual process which takes its starting point from all available evidence related to the crime under investigation. After the criminologist has sorted and organized the facts carefully, he defines the “problem situation,” that is, gaps in his knowledge that need to be filled in order to identify the perpetrator and explain the course of events. Different versions should be formulated that address different aspects of the problem situation. A fundamental requirement of “versions” is that they should be realistic, consistent, and verifiable. Each version leads to different conclusions that can be empirically tested. Building versions provides the investigator with a concrete plan to search for evidence, which can then be used to generate further hypotheses. In general, the ratio of the number of alternative versions should be inversely proportional to the amount of evidence that is available (Stelzer 1978: 159). At the end of the investigation, only one version should survive, which then becomes “categorically certain knowledge.” The creation of “versions” is not just presented as a core fundament of Soviet criminology (Ratinow 1970; Stelzer 1978), but is also frequently mentioned in criminological works on interrogation in the GDR (Scholz & Huß 1976; Hahn & Brunkal 1983; Krüger 1983) as a guiding tool that supposedly leads the interrogator towards the “objective truth” (Stelzer 1984: 30). The creation of “versions” represents an overarching aspect of criminal investigations in GDR criminology. It was not primarily intended to determine the choice between different interrogation tactics, but to guide the investigative process as a whole. More than anything else, the planning and execution of the confrontation in the interrogation room was represented as a matter of psychological expertise, the basics of which are presented in the following.

## Setting the Stage for the Interrogation

According to GDR criminological literature, the ideal investigation of crimes does not start outright with the interrogation. If the suspect has not been arrested yet or does not need to be questioned immediately, the investigation goes through several stages before the interrogator and the defendant face off in the interrogation room. During the preliminary planning phase, the investigator collects as much information as possible about the events that preceded the suspected crime, their legal relevance, as well as the current life circumstances, personality, and potential motives of the suspect (Haase 1980; Grandke 1983; Stelzer 1984). Building upon this, the time and place of the arrest should be planned in detail to avoid unwanted witnesses, spontaneous protests, or the suspect’s escape. Waiting too long, however, carries the risk that the advantage of surprise is lost. The suspect might become suspicious, hide or destroy evidence, prepare for the arrest, and come up with a prepared line of defense. To properly set up the right timing and “angle of attack” of the first interrogation, two components of the crime are differentiated. The “objective” level relates to the time, place, and course of events of the criminal act, including suspected accomplices and witnesses, and all traces which can be analyzed during the preliminary investigation of the crime scene. The “subjective” level is psychological in nature, as the suspect here serves as the primary source of evidence that needs to be analyzed through psychological means: all goals and intentions, attitudes and motives of the defendant, their personality, biography, their past and current life circumstances, as well as their political views and activities fall within this category. Gathering information from colleagues and superiors, friends and family members, and personnel files from the school, place of work, or state authorities all should be included in the planning phase (Lindner 1977). Even though time is often limited and evidence hard to find for the interrogator, it should never be forgotten that preparing and planning is the key to success. When the interrogation begins, it soon turns into a “struggle from which the one who is best prepared emerges victorious” (Stelzer 1984: 97).

Based on all evidence that is gathered during the planning phase, the investigator defines the initial “basic situation,” a key component of the interrogation plan. This plan addresses the “objective” and “subjective” level of the offense, the order of questions, the place and duration of the first interrogation, as well as the use of evidence, documents, and any statements of witnesses that are connected to the crime during the interrogation. Possible reactions by the defendant need to be analyzed beforehand to decide in advance which interrogation tactics should be chosen. In addition, it must be determined how the truth of the suspects’ statements can be verified. One crucial point in this context is the question of whether the accused is likely to cooperate and can be expected to confess or not. If a group of suspects is interrogated, the order of their interrogations must be determined in advance. The common starting point is the “weakest” member of the group who has the highest probability to testify, in order to use the information gathered to exert pressure on the other members of the group. In addition, the interrogator should not just be selected based on his professional competence and experience, but also on his suitability in terms of age, education, or other personal characteristics. Finding a perfect “match” between defendant and interrogator requires a complete and accurate picture of the defendant’s personality, including his family background, relationship status, education, current housing and working circumstances, conflicts at school or at the workplace, previous criminal offences, and any other relevant personal information.

The proper choice of the interrogator(s) also needs to be made during the planning phase, since “not everyone is able to establish the required connection with any person” (Stelzer 1984: 99), regardless of their professional experience and motivation. If the suspect is particularly intelligent and educated, the interrogator should not fall behind in this regard, so he will not be too easily deceived or looked down upon by the defendant. In the case of sexual offenses, female suspects or witnesses should preferably be interviewed by female interrogators, so as not to cause any “additional barriers” (ibid.). The choice of interrogators should not be based solely on their formal qualification and professional experience, but also on “their personal abilities and, to a certain extent, according to their inclinations and in accordance with the personality of the person to be interrogated” (Haase 1980: 50). The interrogator must experience “joy” and “personal success” (ibid.) in his work in order to endure the long and exhausting sessions in the interrogation room without losing sight of the objectives of the investigation.

Altogether, the “planning phase” already went beyond the mere collection of evidence. It was designed to set the stage for the confrontation in the interrogation room by prearranging and orchestrating a diverse set of bureaucratic, legal, material, and social rules and practices. All of these elements were supposed to be combined and adjusted to draw a preliminary picture of the personality of the suspect, including their current motives, attitudes, relationships, and weaknesses. This compilation of evidence, assumptions, and open questions then serves as the starting point for the phase of “first contact,” when the antagonists finally meet.

## First Contact

Once all available evidence concerning the crime and the subject’s personality has been put together and the defendant is under arrest, the planning phase is complete. The interrogation in the narrow sense begins with the “contact phase,” when the interrogator and the defendant face each other for the first time. Initially, the defendant has to be informed about their rights and what charges are raised against them. The suspect is then asked to give their account of the events in their own words, while the interrogator just listens carefully and takes notes. The penal code explicitly grants the defendant the right to provide a “free report” and present any exculpatory evidence at the beginning. According to Römer (1965), the protocol of the first interrogation carries “particular weight,” as the first statement is closest in time to the initial event and has not been influenced by later accounts by the defendant or other witnesses. For the interrogator, the contact phase offers the opportunity to gain a first-hand impression of the accused and test his assumptions about the suspect’s personality, their intellectual and communicational abilities, their emotional state, their plans and intentions, as well as their willingness to cooperate. The interrogator should foster an “atmosphere of trust” (Stelzer 1984: 138) which should calm the suspect, reduce their anxiety, and increase their motivation to testify. Whether the suspect reacts with fear or anger, is silent or talkative, the choice of interrogation tactics must always be constantly evaluated and adapted quickly throughout the procedure. The crucial role of the very first confrontation as a “decisive” moment “for the success of the interrogation” (Gertig & Schädlich 1956) is frequently emphasized. Its “key significance in the context of the first attack” (Haase 1980: 16) is based on the assertion that “the first few minutes can make the difference between success and failure of the interrogation” (Stelzer 1984: 107). Although the interrogator has the advantage of surprise and the initiative on his side, he must also be aware that any misjudgment can have serious consequences for the rest of the investigation. Missing out crucial details at the planning stage or choosing the wrong interrogation tactic may lead to a total refusal to testify or to false statements, which require a revision of the interrogation tactics or a replacement of the interrogator. If the “contact phase” was successful and a free account of the events was given by the suspect, the second phase of the interrogation follows, wherein individual statements are systematically evaluated by the interrogator.

## The Dissection of Statements

Over the years, the criminologists working at Humboldt University put together an extensive list of psychological traits and processes that allegedly determine individual statements during the interrogation. Based on the works of Rubinstein (1959) and Ratinow (1970: 222–42), individual statements were to be analyzed by following a threefold sequence of perception, memory, and reproduction. All three stages are influenced by various biological, situational, and individual factors on the objective and subjective level, which altogether determine the accuracy and credibility of any given statement: poor lighting, distracting noises, and a lack of attention can diminish the ability of defendants or witnesses to reproduce the details of an event. Alcohol consumption, overexcitement, tiredness, or neurological diseases can have a negative effect on their memory. Lack of language skills, distraction, or fear of the interrogator may decrease their ability to give a coherent account of events. Human attention is fundamentally selective, memory is unavoidably incomplete and distorted, and the ability to verbalize thoughts and ideas is generally very limited (Stelzer 1984: 76–85). The interrogator must also be aware of the effects of his own behavior. Exhaustive questioning over many hours can lead to a “deterioration in the ability to testify,” which does not necessarily mean that the suspect does not want to cooperate. Referring to Hermann Ebbinghaus’s classical work on the psychology of memory, the audience is informed that the curve of recall is steep at first and then flattens out over time, meaning that people tend to forget most information in the beginning, but retain some bits for a longer time. This makes it all the more important to plan the contact phase as thoroughly as possible and proceed while memory is still fresh (Stelzer 1984: 89). The more time goes by between the event and the report of the defendant or witness, the more easily memory can be distorted or fragmentary. Frequent recounting of events can also turn out as problematic, as the account becomes a reproduction of a reproduction rather than of what was perceived initially, and interrogators who expect literal repetitions of the same account risk reinforcing reiterations of previous statements rather than true recollections of events. Another common source of error on the side of the interrogator comes from the ignorance of the power of suggestion. As most interrogations are “interspersed with unintentional suggestive factors” (Stelzer 1984: 86), the interrogator must always keep in mind how he phrases his questions, how they could have influenced the answer, and how he reacts to the answers of the defendant.

In order to keep an overview of this multitude of psychological elements of the investigative process, Römer suggests adopting a developmental and holistic perspective. According to this view, each statement must be understood in its context and in light of the preceding events. The mental processing and interpretation of what was perceived can go on for a long period of time, running through different phases and stages, wherein the “oral testimony to the investigative body represents only the last link in the chain” (Stelzer 1984: 91). Therefore, isolated statements should always be analyzed in their respective context, that is, in their temporal sequence as well as in relation to the “whole of the personality,” as the “origin of the statement cannot be separated from the person making it” (Stelzer 1984: 75). The interrogator needs to understand that there is a “unity of the personality of the person testifying and their statement” (Stelzer 1984: 90). This principle represents a “core element to evaluate a statement” (Stelzer 1984: 75–76). Individual statements are analyzed not only to assess the credibility of the testimony, but also to determine whether the accused is telling the truth, deliberately lies, or is simply inaccurate or mistaken.

With regard to the reliability of individual statements, the “bourgeois” psychology of testimony is frequently criticized in Soviet criminology. Early-twentieth-century psychologists such as William Stern or Otto Lipmann, who suggested that accurate testimony is an exception and not the rule, were discounted as representatives of a system of “arbitrary punishment through the bourgeois justice system” (Ratinow 1970: 218). According to the socialist criminologists, Stern’s general assessment of the unreliability of witness testimony was supposedly based on a reductionist analysis of testimony in the “isolated psychological experiment” (Stelzer 1984: 71). The temporary success of the psychology of testimony in the early twentieth century was supposedly based on its service to class interests. In the eyes of socialist criminologists, Western interrogation studies demonstrate the hostile relationship between the capitalist state and the working class, wherein citizens tend to lie to the police or in court for a good reason: their “well-founded mistrust in the practices of the police and the judiciary” of the bourgeois state and its “manipulation of opinions … undoubtedly have a negative effect on testimony” (Stelzer 1984: 69). GDR criminologists saw a continuation of these “anti-human practices” in the USA, where they are used as “prototypes of a psychology adapted to class justice,” and the criminal investigator acts like an “inquisitor without any principles.” In opposition to that, the socialist investigator uses psychological knowledge merely as an “important aid to practically realize socialist humanism” (Stelzer 1984: 72). The fixation on the “negative effect of emotions on witness accounts” is a characteristic of bourgeois science, a symptom of an “antagonistic and competitive system” (Stelzer 1984: 92). Socialism, on the other hand, would produce completely different, positive feelings among the members of the working class, such as identification with the authorities, hatred towards the class enemy, and enthusiasm for work. The longer the interrogation goes on, the more it goes beyond the mere collection and analysis of information. The interrogation room is much more than a controlled environment to gather information and produce confessions. For the socialist interrogator, interrogation is not only a practice of gathering evidence, but also an instrument of education: “Interrogation is not only about a past or present state, but also about future behavior” (Stelzer 1984: 67). As this connection between interrogation and education plays an outstanding role in GDR criminology, it is highlighted below as the final psychological element of the socialist approach towards interrogation.

## Interrogation as Education

The first phase of compiling the interrogation plan and setting up the first contact was designed to systematically gather as much information as possible about the offense, as well as the personality and motives of the suspect. Once the questioning phase begins, the scope and complexity of the interrogation increase significantly, transcending the mere function of gathering evidence. As a communicative, reciprocal act, the interrogation always needs to be understood as a relational phenomenon. In fact, several relationships can influence the course of the interrogation: the relationship of the suspect with their accomplices, relatives, or colleagues can play a decisive role in their willingness to testify or confess, even if the suspect has no options to communicate with the outside world. Fear of accusing or disappointing others, feelings of shame, or the expectation of later punishment might reduce the willingness to testify. To decrease these unwanted, inhibiting factors, the interrogator needs to focus on his own relationship with the defendant, a relationship which grows and develops the longer the interaction goes on: the “relationship established by the interrogator during the contact phase determines the entire interrogation” (Grandke 1983: 36).

The more time and effort the interrogator invests in getting to know the defendant’s personality, the better the chance of improving what is initially a rather confrontational and conflictual relationship. The defendant might feel surprised, overwhelmed, defenseless, threatened, and frightened when exposed to the power of the state. As the defendant spends days, weeks, and months between the prison cell and the interrogation room, their motives and emotions change over time. Even if they refuse to testify in the beginning, opportunities might come up for “the conscious activation and utilization of such emotions in the respondent that encourage truthful statements” (Stelzer 1984: 123). Feelings of isolation, loneliness, and despair, combined with a growing attachment to the interrogator, the only person with whom they communicate for such a long time, can be used to improve the relationship between the antagonists. The interrogator should use this situation in a way that creates the impression that the defendant’s “problems and his situation … are also met with a certain understanding” (Stelzer 1984: 124).

The “educational” aspect of interrogation is frequently underlined in the criminological literature of the GDR. In addition to gathering evidence for the court hearing, the interrogation was also presented as means to influence the suspect. The confession supposedly represented “one of the most important prerequisites for a successful education and rehabilitation of the offender” (Stelzer 1984: 129). The study of the suspect’s personality during the preparatory phase is not only necessary to solve the crime, but also represents a “focused educational intervention” (Stelzer 1984: 99). When groups are arrested, the correct choice of interrogation tactics can help “dismantle the prevailing value systems,” undermine group solidarity, and turn group members against each other. In these cases, the interrogation fulfils a potentially “important educational role” (Stelzer 1984: 142) that begins long before a judgement is made by the court. This aspect of questioning is important not only for children and adolescents, but for defendants of all ages: “Through the interrogation, the criminologist actively and directly influences the process of raising awareness to comply with the norms of social coexistence in the future” (Lindner 1977: 5). The ultimate goal of the interrogation needs to be understood in connection with the supposed “educational influence on the offender” (Haase 1980: 6). As he confesses, the defendant should prove his sense of responsibility, remorse, and feeling of guilt. However, this transformation can only succeed if the interrogator gains the trust of his counterpart, who should recognize him as an ally on his way back outside. The criminologist should be a “good listener” and he needs to keep in mind that he not only represents the authority of the state, but should also act as a “counsellor and helper” (Grandke 1983: 38). Only those who truly understand the thoughts, feelings, and biography of the defendant will be able to reach them on an emotional level. When the interrogation has ended, the defendant should want to become a better citizen after receiving their just punishment. This change in attitude could already be demonstrated during the interrogation, for example by naming other offenders, helping to solve other crimes, or agreeing to cooperate with the authorities in the future. This practice of “turning round” defendants was a specialty of members of the State Security in the interrogation of political prisoners, who would later on stay in touch with their “helpers” after release.

## Conclusion

At first sight, the science or “art” (Stelzer 1984: 72) of interrogation, as it is presented in the criminological literature from the GDR, appears primarily as an attempt to systematize, standardize, and professionalize the work of experts from the jurisdiction, the police, and the State Security. The growth and development of this body of knowledge from the 1960s onwards was paralleled by an attempt to elevate criminological training to academic standards. On the organizational level, psychology benefited from this process through the integration of psychologists into the Criminological Section and the establishment of a separate study program in forensic psychology. However, the dissolution of this study program after just two years and the failure to establish forensic psychology as an independent discipline showed that the interest in psychological knowledge from other professions also had its limits. The value of psychological knowledge was measured by its presumed contribution to making criminological practices more effective, but that did not mean that the training of psychological experts was seen as a necessary part of this endeavor. Accordingly, it was not psychological experts, but professionals from law and the police, such as Ehrenfried Stelzer and Axel Römer, who frequently resorted to psychology in their works on interrogation. Therefore, Eghigian’s assessment of a “psychologization” (2004) of GDR criminology might need to be reconsidered in the light of the material described above: it was not so much the handful of psychologists who actively transformed the field, but rather a broader change that took place within criminology and post-Stalinist socialism that led to a selective adoption of psychological knowledge in the training of interrogators.

On a technical level, the socialist approach towards interrogation was not unique. Practices such as the careful preparation of the interrogation plan, the preliminary exploration of the suspect’s personality, the analysis of the social and biographical background of the defendant, and the search for entry points and weaknesses to facilitate a communicative basis have already been described as early as in the late eighteenth century (Schuman 2016: 107) and they were well known in Germany before World War II (Lück & Niehaus 2007: 170). These practices can be interpreted as a typical characteristic of modern criminalistic work since it became part of a bureaucratized institutional network that existed before and outside of the socialist realm (Reichertz & Schneider 2007; Schumann 2016). The analysis of the individual steps of the interrogation process and the corresponding psychological factors by the Soviet criminologists was driven less by a pursuit of theoretical synthesis than by the bureaucratic requirements of the Code of Criminal Procedure, as well as the requirements of the police authorities, who had to adapt their practices to the conditions of the post-Stalinist era (Gieseke 2021). As they turned away from physical violence, they were searching for other means to fulfill their duties in the interrogation room. Nevertheless, the criminologists repeatedly pointed out that there are no universal “recipe-like recommendations” (Haase 1980: 96) which could replace practical experience and trained intuition. Every interrogation “requires an individual approach and an individual assessment … There are no easy solutions, no tricks or gimmicks that can be used to achieve success quickly and everywhere” (Stelzer 1984: 72). The ambivalence between the claim that the “art” of interrogation could be taught, on the one hand, and the need for “creative,” spontaneous, and intuitive thinking, on the other, could never be fully resolved and left the ultimate responsibility for the outcome of the interrogation to the investigator.

So, did socialists interrogate differently? The fact that GDR criminologists answered this question in the affirmative is not too surprising, since they aimed to distinguish their “humanistic” approach from the “manipulative” techniques of the bourgeois class enemy. In their view, the growing interest in psychological knowledge for criminal investigations was “international,” but its use “in capitalist and socialist countries … [was] based on completely opposing class interests.”^18^ From the 1960s onwards, GDR criminologists repeatedly emphasized that using physical violence, or threatening to do so, is not a feasible solution, arguing that the “ethics of the socialist investigator” do not allow the use of “physical violence, false promises, threats” or “bluffs” (Haase 1980: 92). It is a different question, however, to what extent officers followed these recommendations. Many accounts of former prisoners attest to the fact that what happened in the interrogation room was often experienced very differently (Fritzsch 1993; Jablonski 1997; Knabe 2007).

The ideological alignment of socialist interrogation is most evident in the principle of “partiality,” which instructs the interrogator to understand his position as a representative and defender of the socialist state who has to evaluate the defendant’s behavior and personality according to socialist laws and norms. In the reformed, post-Stalinist context, his methods should not build upon terror and intimidation, but on a more nuanced, bureaucratic, and scientific approach. Beyond the examination of the circumstances of the offense and the personality of the defendant, the core specificity of socialist interrogation lies here: to bring about a profound change in the attitudes, long-term goals, thoughts, and feelings of the offender, a transformation that goes way beyond a superficial or short-term adjustment to the current situation. If, when, and where this goal was also achieved in practice, and whether similar practices against political prisoners were also used in other contexts beyond the socialist sphere must be discussed elsewhere (Kenney 2017; Michels & Wieser 2018). From 1962 onwards, there was also the possibility of West Germany buying out political prisoners from the GDR. Interrogators and defendants often had this option in mind, which could render any measures of “reeducation” obsolete. Furthermore, some oppositional groups were aware of the psychological training of their opponents and also prepared themselves for the expected confrontation in the interrogation room (Wieser 2025).

Setting aside these limitations and unanswered questions, we can underline the observation that the central role of interrogation practices in the socialist sphere never diminished from Koestler’s time until the end of the Cold War. While the ultimate aim of the interrogation, the production of confessions, remained unchanged, the concrete steps to achieve this goal became a matter of concern for a growing number of criminological experts during and after the 1960s. Psychological knowledge was only one element among many others in this context, but its continuous presence at the Criminological Section of Humboldt University and at the Juridical Academy Potsdam shows that the socialist interrogator was expected to be properly prepared on a psychological level before he entered the arena of the interrogation room.

## Footnotes

1 Humboldt Universität zu Berlin, Universitätsarchiv, Sektion Kriminalistik, Nr. 246, Statistik Studenten der Sektion Kriminalistik, 1978–1988.

2 Humboldt Universität zu Berlin, Universitätsarchiv, Juristische Fakultät, Institut für Kriminalistik, Nr. 102, Ordnung und Statut der FR Kriminalistik und des Instituts für Kriminalistik der HU, 1962: 7.

3 Humboldt Universität zu Berlin, Universitätsarchiv, Sektion Kriminalistik Grundsatzdokumente, Nr. 67, Altersstruktur und wissenschaftliche Mitarbeiter, 1971–1979.

4 See Endnote 1

5 Humboldt Universität zu Berlin, Universitätsarchiv, Sektion Kriminalistik, Nr. 247, Statistik – Lehrkörper und Studenten der Sektion Kriminalistik, 1976–1984.

6 Humboldt Universität zu Berlin, Universitätsarchiv, Sektion Kriminalistik, Nr. 114/1, Studienvoraussetzungen, Studienpläne, 1979–1986.

7 Humboldt Universität zu Berlin, Universitätsarchiv, Sektion Kriminalistik, Nr. 1203, Einschätzung von Axel Römer, n.d.

8 Humboldt Universität zu Berlin, Universitätsarchiv, Sektion Kriminalistik, Nr. 35, Studienorganisation, 1972–1979. Konzeption, 12.6.1972.

9 Humboldt Universität zu Berlin, Universitätsarchiv, Sektion Kriminalistik, Nr. 24, Lehrprogramme und Vorlesungsplanung, 1964–1975. Vorbemerkungen zum Lehrprogramm für die Fachausbildung von Diplompsychologen der Fachstudienrichtung forensische Psychologie, 7.6.1972: 85.

10 Stasi-Unterlagen-Archiv, MfS, AKK, Nr. 13818/76.

11 Humboldt Universität zu Berlin, Universitätsarchiv, Sektion Kriminalistik, Nr. 186, Entwicklung des Fachbereichs forensische Psychologie 1969–1987. Aktenvermerk zur Problematik Prof. Dr. sc. Reiner Werner, 20.8.1973.

12 Humboldt Universität zu Berlin, Universitätsarchiv, Sektion Kriminalistik, Nr. 100, Sektionsrat, 1968–1974. Protokoll des Sektionsrates der Sektion, 8.11.1973: 5.

13 Ibid.: 13

14 Humboldt Universität zu Berlin, Universitätsarchiv, Sektion Kriminalistik, Nr. 100, Sektionsrat, 1968–1974. Protokoll des Sektionsrates der Sektion, 15.12.1972: 4.

15 See Endnote 3

16 Humboldt Universität zu Berlin, Universitätsarchiv, Sektion Kriminalistik, Nr. 440, Die Vernehmung, n.d.: 6.

17 Ibid.

18 Ibid.: 9.
